# Annexin A1 Bioactive Peptide Promotes Resolution of Neuroinflammation in a Rat Model of Exsanguinating Cardiac Arrest Treated by Emergency Preservation and Resuscitation

**DOI:** 10.3389/fnins.2019.00608

**Published:** 2019-06-14

**Authors:** Qing Ma, Zhiquan Zhang, Jae-Kwang Shim, Talaignair N. Venkatraman, Christopher D. Lascola, Quintin J. Quinones, Joseph P. Mathew, Niccolò Terrando, Mihai V. Podgoreanu

**Affiliations:** ^1^Systems Modeling of Perioperative Organ Injury Laboratory, Department of Anesthesiology, Duke University, Durham, NC, United States; ^2^Neuroinflammation and Cognitive Outcomes Laboratory, Department of Anesthesiology, Duke University, Durham, NC, United States; ^3^Center for Translational Pain Medicine, Duke University, Durham, NC, United States; ^4^Department of Anesthesiology and Pain Medicine, Yonsei University College of Medicine, Seoul, South Korea; ^5^Departments of Radiology and Neurobiology, Duke University, Durham, NC, United States; ^6^Duke-UNC Brain Imaging and Analysis Center, Duke University, Durham, NC, United States; ^7^Department of Anesthesiology, Duke University, Durham, NC, United States

**Keywords:** sirtuins, autophagy, apoptosis, neuroprotection, HMGB1

## Abstract

Neuroinflammation initiated by damage-associated molecular patterns, including high mobility group box 1 protein (HMGB1), has been implicated in adverse neurological outcomes following lethal hemorrhagic shock and polytrauma. Emergency preservation and resuscitation (EPR) is a novel method of resuscitation for victims of exsanguinating cardiac arrest, shown in preclinical studies to improve survival with acceptable neurological recovery. Sirtuin 3 (SIRT3), the primary mitochondrial deacetylase, has emerged as a key regulator of metabolic and energy stress response pathways in the brain and a pharmacological target to induce a neuronal pro-survival phenotype. This study aims to examine whether systemic administration of an Annexin-A1 bioactive peptide (ANXA1sp) could resolve neuroinflammation and induce sirtuin-3 regulated cytoprotective pathways in a novel rat model of exsanguinating cardiac arrest and EPR. Adult male rats underwent hemorrhagic shock and ventricular fibrillation, induction of profound hypothermia, followed by resuscitation and rewarming using cardiopulmonary bypass (EPR). Animals randomly received ANXA1sp (3 mg/kg, in divided doses) or vehicle. Neuroinflammation (HMGB1, TNFα, IL-6, and IL-10 levels), cerebral cell death (TUNEL, caspase-3, pro and antiapoptotic protein levels), and neurologic scores were assessed to evaluate the inflammation resolving effects of ANXA1sp following EPR. Furthermore, western blot analysis and immunohistochemistry were used to interrogate the mechanisms involved. Compared to vehicle controls, ANXA1sp effectively reduced expression of cerebral HMGB1, IL-6, and TNFα and increased IL-10 expression, which were associated with improved neurological scores. ANXA1sp reversed EPR-induced increases in expression of proapoptotic protein Bax and reduction in antiapoptotic protein Bcl-2, with a corresponding decrease in cerebral levels of cleaved caspase-3. Furthermore, ANXA1sp induced autophagic flux (increased LC3II and reduced p62 expression) in the brain. Mechanistically, these findings were accompanied by upregulation of the mitochondrial protein deacetylase Sirtuin-3, and its downstream targets FOXO3a and MnSOD in ANXA1sp-treated animals. Our data provide new evidence that engaging pro-resolving pharmacological strategies such as Annexin-A1 biomimetic peptides can effectively attenuate neuroinflammation and enhance the neuroprotective effects of EPR after exsanguinating cardiac arrest.

## Introduction

Exsanguinating hemorrhage, leading to cardiac arrest and multiple organ failure, remains the most common cause of death among trauma patients without traumatic brain injury, and neurologic outcomes in survivors are poor. An new therapeutic paradigm – termed EPR – involves rapidly cooling victims of exsanguinating cardiac arrest to deep or profound hypothermia levels (≤20°C), in an effort to extend ischemic time, maintain organ viability during severe shock, allow operative repair of injuries and resuscitation, and ultimately improve survival and preserve neurological function (Tisherman et al., 2017). Following initial characterization of EPR, a growing body of proof-of-concept large animal studies have investigated the optimal depth and rate of cooling, duration of EPR, and physiological conditions prior to induction of EPR (Rhee et al., 2000; Alam et al., 2002, 2004, 2005, 2006a,b; Wu et al., 2006). Rapid cooling and subsequent controlled resuscitation and rewarming is accomplished through the use of CPB (Sailhamer et al., 2007; Alam et al., 2008). The development of rodent models of HS and EPR enabled characterization of key cellular and molecular changes involved in exsanguination cardiac arrest (Drabek et al., 2007a,b) – chief among those are neuroinflammation and apoptosis – which constitute targets for adjunctive therapies (Han et al., 2008; Alam et al., 2010).

Neuroinflammation is a complex immune response commonly observed following acute brain insults such as HS and associated warm ischemia, ischemia-reperfusion injury (I/RI), therapeutic hypothermia, exposure to CPB, and post-cardiac arrest syndrome. Multiple rodent models of surgical trauma, cardiac arrest and resuscitation have identified upregulation of pro-inflammatory cytokines and inflammatory mediators in both peripheral tissues and the central nervous system (CNS) (Terrando et al., 2010a, 2011). Acute neuroinflammation is characterized by increased cytokines, such as interleukin-1β (IL-1β), tumor necrosis factor-α (TNF-α), HMGB1 and their cognate receptors ultimately leading to neuron-glia dysfunction and blood–brain barrier impairments (Cao et al., 2010; Terrando et al., 2010b, 2011; He et al., 2012; Liu and McCullough, 2013; Wohleb et al., 2014; Xiang et al., 2016; Skvarc et al., 2018). Oxidative stress and mitochondrial dysfunction converge with the neuroinflammatory pathway, creating a positive feedback loop (Netto et al., 2018). Collectively, these mechanisms actively contribute to neuronal death and cognitive impairment. There is evidence that the neuroinflammatory *milieu* persists following surgical trauma, HS, I/RI, and resuscitation from cardiac arrest because is not efficiently controlled by endogenous anti-inflammatory mechanisms, thereby contributing to secondary injury, CNS dysfunction, and neuronal network hyperexcitability (Tahsili-Fahadan et al., 2018). Despite improvements in resuscitation science for victims of HS cardiac arrest, including EPR, total body cooling is technically and logistically challenging and the development of effective therapies to decrease morbidity and improve long-term neurologic outcomes following exsanguinating cardiac arrest remains a critical need.

Dysfunction of inflammation-resolving pathways have been described in experimental rodent trauma models, leading to exaggerated post-injury cognitive decline (Su et al., 2013). In recent years, a number of lipid mediators such as resolvins, lipoxins, and maresins have begun to receive attention as possible resolvers of neuroinflammation (Terrando et al., 2013; Vacas et al., 2013). Resolvins act to block neutrophil and monocyte migration and reduce the oxidative burst of neutrophils (Serhan et al., 2007; Perretti et al., 2015). Similarly, the glucocorticoid-regulated protein Annexin A1 and its peptide mimetics display pro-resolution effects, and have been shown to mitigate cerebral I/RI by attenuating neuroinflammation in many experimental models (Ansari et al., 2018) including cardiac surgery with deep hypothermic circulatory arrest (Zhang et al., 2017). The aim of this study was to determine the neuroprotective efficacy of a bioactive Annexin A1 short peptide (ANXA1sp) in a small animal model of exsanguination cardiac arrest and EPR. We hypothesized that ANXA1sp treatment would attenuate neuroinflammation and neuronal cell death, in part through upregulation of the mitochondrial protein deacetylase SIRT3 and downstream cytoprotective pathways.

## Materials and Methods

### Animals

The experimental protocol was approved by the Duke University Animal Care and Use Committee. All procedures met the guidelines of the National Institutes of Health for animal care (Guide for the Care and Use of Laboratory Animals, Health and Human Services, National Institute of Health Publication No. 86-23, revised 1996). Adult male Sprague–Dawley rats (age 13–15 weeks; weight 400–450 g; Charles River Laboratories, Wilmington, MA, United States) were housed (two animals per cage) in a 12-h light-dark cycle environment with free access to food and water. Rats were acclimated for at least 1 week before starting the experiment.

### Surgical Preparation for Experimental EPR

**FIGURE 1:**
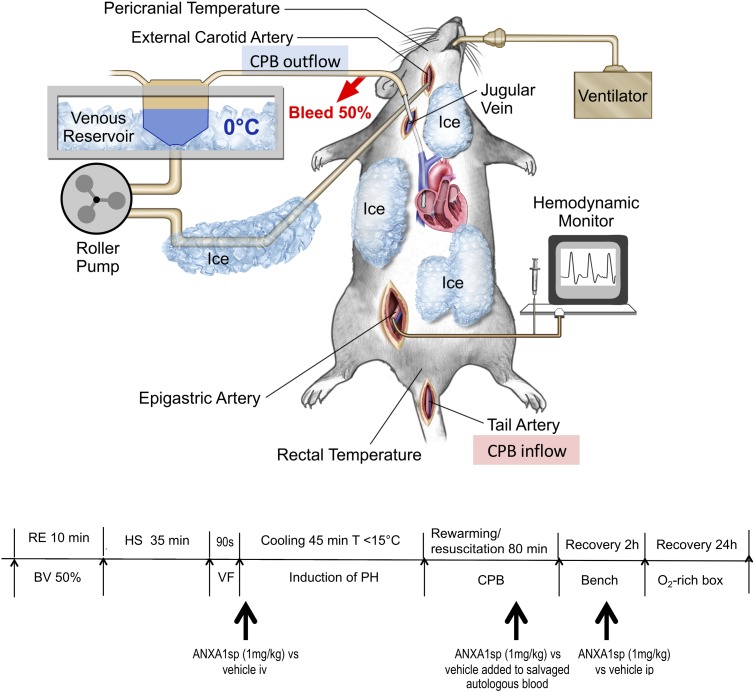
Experimental model of exsanguination cardiac arrest including rapid hemorrhagic shock followed by 90 s of fibrillatory arrest, induction of profound hypothermia (<15°C), resuscitation and rewarming on CPB (emergency preservation and resuscitation, EPR) – diagram of surgical model and experimental timeline. RE, rapid exsanguination; BV, blood volume; HS, hemorrhagic shock; VF, ventricular fibrillation; PH, profound hypothermia; CPB, cardiopulmonary bypass; ANXA1sp, Annexin A1 short peptide.

Experimental procedures are summarized in Figure 1. Fasted rats were anesthetized with isoflurane, intubated, and mechanically ventilated to maintain the arterial PaCO_2_ between 35 and 45 mmHg. Anesthesia was provided with isoflurane (1.5–2%) with an oxygen and air mixture at a FiO_2_ of 0.5. Bupivacaine 0.25% was injected at the operative sites to achieve local anesthesia. Routine physiologic parameters, and rectal and pericranial [hypodermic needle probe (Omega^®^, Stamford, CT, United States) placed beneath the temporalis muscle] temperatures were monitored. The right caudal epigastric artery was cannulated with a polyethylene catheter (PE10, Clay Adams, Sparks, MD, United States) and used for systemic mean arterial pressure (MAP) monitoring. The tail artery was cannulated with a 20-gauge catheter, which served as blood sampling and inflow CPB cannula. The right internal jugular vein was cannulated with a customized 4.5-F multi-orifice catheter advanced into the right atrium and used for withdrawing blood and venous out-flow CPB cannula. The external carotid artery was cannulated with a polyethylene catheter (PE50; Clay Adams, Sparks, MD, United States) advanced into the aortic arch and used for induction of profound hypothermia by flushing ice-cold normal saline. During surgical preparation, normothermia was maintained using a heat lamp.

#### Induction of Hemorrhagic Shock and Ventricular Fibrillation

After a 30-min equilibration period, rapid exsanguination (50% blood volume, 0.034 ml/g body weight, 12–15 ml of blood) over 10 min was performed via the jugular venous cannula and MAP was allowed to drop below 20 mmHg. For the next 35 min (simulating pre-hospital transport time), the MAP remained <20 mm Hg without any resuscitative efforts HS. The shed blood was salvaged in a 20 ml syringe prefilled with 2 ml citrate phosphate dextrose solution (Fenwal Inc., Lake Zurich, IL, United States) and stored at 4°C for later transfusion/resuscitation. At the end of HS, ventricular fibrillation (VF) was induced by transesophageal burst pacing at 50 V (S48 stimulator, RI, United States) for 90 s, and confirmed electrocardiographically and echocardiographically (Phillips Sonos7500 system, Andover, Mass 01810). During HS, anesthesia was maintained using 0.4% isoflurane. No neuromuscular blocker was administered until just prior to VF. A dose of vecuronium bromide (0.1 mg/kg) was given intravenously to prevent muscle contraction during VF and cooling.

#### Induction of Profound Hypothermia

After the induction of VF, the animals were rapidly cooled to a core temperature of 10–15°C for 45 min by flushing ice-cold solution [total volume 45 ml = 40 ml normal saline + 2 ml 5% human plasma protein fraction (Plasmanate^®^, Grifols Therapeutics, Inc., NC, United States) + 3 ml 8.5% Sodium Bicarbonate] into the ascending aorta/aortic arch and returning from right jugular vein using a peristaltic pump (Masterflex, model 770201-60, Cole-Parmer, Vernon Hills, IL, United States) at rate of 20 ml/kg/min (T208 transonic volume flow meter, Transonic Systems Inc. Ithaca, NY, United States). A water bath with ice water as well as topical cooling with ice bags was also applied.

#### Resuscitation and Rewarming With Cardiopulmonary Bypass

Following 45 min of profound hypothermia, rats were rewarmed and resuscitated using CPB. The CPB circuit consisted of a peristaltic pump (MasterflexC, Cole-Parmer, Vernon Hills, IL, United States), a custom-designed oxygenator, and a venous reservoir, as previously described (Bartels et al., 2014; Shim et al., 2014; Zhang et al., 2017). The reservoir was primed with 3 ml of 6% hydroxyethylstarch 130/0.4 and 2 ml of previously shed and salvaged blood. Heparin (200 IU) and vecuronium bromide (0.1 mg/kg) were added to the venous reservoir. CPB was initiated at a flow rate of 20–30 ml/min, which was gradually increased to 50–60 ml/min upon reaching 33–34°C. The gradient between the water bath and core body temperatures was not allowed to exceed 10°C. Once core temperature increased from 10 to 34°C, all salvaged blood was gradually re-transfused to keep up with the increased oxygen demand. The animals were rewarmed for 80 min until core temperatures of 34°C were achieved; subsequently, CPB was terminated. Rewarming rate was maintained at approximately 0.4°C/min. Acid-base abnormalities were corrected as needed. During rewarming, MAP was kept above 50 mmHg once core temperature reached >30°C using intermittent administration of epinephrine.

After decannulation, animals were kept ventilated for 2 h under anesthesia with 0.5–1% isoflurane (core temperature 36–37°C). To increase the hematocrit value to greater than 30%, the remaining autologous blood in the CPB circuit was collected, concentrated by centrifugation (3000 rpm for 5 min), and re-transfused. Heparin-induced anticoagulation was not reversed and allowed to dissipate spontaneously. Routine blood gas analysis was conducted serially (GEM Premier 3000 analyzer, Instrumentation Laboratory, Bedford, MA, United States). After spontaneous ventilation had resumed, animals were extubated and allowed to recover in an oxygen-enriched and humidified environment for 24 h, with free access to water and food.

To harvest the brain, 24 h after EPR rats were re-anesthetized, intubated, and mechanically ventilated. One sample of brain tissue was immediately fixed in 10% buffered formalin and paraffin-embedded for immunostaining. The remaining brain tissue was frozen in liquid nitrogen and stored at -80°C until further use. Blood samples were also collected before and after induction of HS and at 0–120 min and 24 h after CPB and stored at -80°C until analysis. Naïve rats were sacrificed under 5% isoflurane.

### Drug Treatments

Annexin A1 biomimetic tripeptide (ANXA1sp or Ac-QAW, Ac = acetyl, MW = 445.47 Da) was synthesized and purified (>98% purity) by GenScript (Piscataway, NJ, United States). The peptide was suspended in 100% DMSO. For experiments, this stock solution was diluted in saline to a final dose of 1 mg/kg ANXA1sp and a concentration of 1% DMSO in saline as vehicle (control). ANXA1sp treatment solutions were freshly prepared immediately before use.

Rats were randomly assigned to three groups (*EPR + ANXA1sp*, *n* = 14; *EPR*+ *vehicle*, *n* = 15; *naïve controls*, *n* = 3) and terminated for histologic and biochemical analyses at 24 h after EPR. Rats received ANXA1sp (1 mg/kg iv) or vehicle (1% DMSO iv) in 1 ml saline immediately after induction of VF, then again at 1 h after CPB. Additionally, blood salvaged during exsanguination HS was also treated *ex vivo* with ANXA1sp 1 mg/kg or 1% DMSO and re-transfused during CPB rewarming and resuscitation (Figure 1). All treatments were administered in a blinded manner.

### Cell Death Assessment

Apoptosis was determined by terminal deoxynucleotidyl nick-end labeling (TUNEL) per assay manufacturer’s protocol (Roche Diagnostics, Indianapolis, IN, United States). Briefly, sections of paraffin-embedded brain tissue samples (5 μm thick) were deparaffinized using xylene and descending grades of ethanol, and pretreated with microwave radiation (350 W, in 200 mL of 0.1 M Citrate buffer, pH 6.0) for 5 min. Tissue sections were then incubated with terminal deoxynucleotidyl transferase (TdT) for 1.5 h at 37°C and then rinsed with PBS. Slides of five representative areas of the retrosplenial and posterior parietal cortex and CA1 area of the hippocampus were mounted using UltraCruz^TM^Mounting Medium with DAPI (Santa Cruz Biotechnology, Santa Cruz, CA, United States). Negative controls were incubated in label solution without TdT. A separate set of brain tissue sections was stained with acid fuchsin-celestine blue to identify possible necrotic cells. Cell counting was performed in a blinded manner across five representative areas of the cerebral cortex and CA1 areas using fluorescence microscopy (Leica DM IRB, Germany) with a 20×/0.4 PH objective at 1.5-fold magnification. Data obtained in every field were added together to make a final data count for each slide and expressed as percentage of total cell number within the relevant fields.

### Western Blots

Frozen brain samples were homogenized and protein quantified by BCA assay (Thermo Fisher Scientific). Western blotting was performed using SDS–PAGE 4–20 and 8–16% gels gradient gels (Bio-Rad) with the following antibodies: rabbit anti-HMGB1 (Bioss Antibodies Inc., Woburn, MA, United States), rabbit polyclonal antibodies against SIRT3 (28-kDa isoform, Cell Signaling and Abcam), FOXO3a (Cell Signaling), Mn-SOD (Santa Cruz), cleaved caspase-3 (Cell Signaling), Bax and Bcl-2 (Santa Cruz), rabbit anti-LC3B (cell signaling), mouse anti-p62/SQSTM1 (R&D Systems, Minneapolis, MN, United States), and rabbit monoclonal antibody against GAPDH (Cell Signaling Technology, Boston, MA, United States). The bands were detected by Super-signal West Dura Extended Duration Substrate (Thermo Scientific Fisher, Rockford, IL, United States). Band intensities of HMGB1, SIRT3, FOXO3a, Mn-SOD, cleaved caspase-3, Bax, Bcl-2, LC3II, p62/SQSTM1 were all normalized with a GAPDH loading control.

### Cytokine Measurements

Concentrations of IL-10, IL-6, and TNFα in brain homogenates were measured using rat-specific ELISA kits per manufacturer’s protocol (Thermo Fisher Scientific, Grand Island, NY, United States). Brain homogenates were separated by centrifugation at 14,000 *g* for 10 min at 4°C to remove cellular debris. In addition, left ventricular myocardial concentrations of IL-6 and TNFα were analyzed by ELISA. Change in absorbance in every well was detected at 450 nm on a microplate reader. All measurements were performed in triplicate.

### Confocal and Fluorescence Microscopy

After deparaffinization, brain tissue sections were treated with 10 mM citrate buffer (pH 6.0) for antigen retrieval. After blocking with 10% normal goat serum at RT for 1 h, the sections were incubated with rabbit anti-SIRT3 antibody (1:300) and mouse anti-COXIV (1:500, Santa Cruz Biotechnology, Santa Cruz, CA, United States), or rabbit anti-LC3B (1:400) and mouse anti-p62/SQSTM1 (1:400), respectively, at 4°C overnight. The sections were then incubated with Alexa Fluor 488-conjugated goat anti-rabbit IgG (1:500; Invitrogen, Carlsbad, CA, United States) and Alexa Fluor 550-conjugated goat anti-mouse IgG (1:500; Invitrogen, Carlsbad, CA, United States) at RT for 1 h. After washing with PBS, slides were prepared and mounted using UltraCruz^TM^ Mounting Medium with DAPI (Santa Cruz Biotechnology, Santa Cruz, CA, United States) to detect nuclei. Images were captured on a Leica SP5 confocal microscope (Leica Microsystems, Germany) using a 40×/1.25–0.75 Plan APO oil objective, and the images were analyzed by NIH ImageJ software (version 1.51).

To assess biodistribution of systemically administered ANXA1sp, rats were anesthetized, intubated and mechanically ventilated, and the tail vein cannulated as described above. Fluorescein isothiocyanate (FITC)-conjugated ANXA1sp (FITC-QAW, MW = 905.48, >98% purity) was synthesized and purified by GenScript (Piscataway, NJ, United States) and injected via the tail vein (0.5 mg/kg). Brain tissue was harvested at 1 h after injection, fixed with 10% neutrally buffered formalin and embedded in paraffin. The 5 μm sections were deparaffinized and evaluated by fluorescence microscopy as above.

### Neurologic Evaluation

On postoperative day 1, rats underwent standardized functional neurologic testing by an observer blinded to group assignment, using an established neurologic scoring system that evaluates motor deficit (Homi et al., 2010). Briefly, rats were first placed on a 35 × 31 cm screen (grid size 0.6 × 0.6 cm) that could be rotated from horizontal (0°) to vertical (90°). The length of time that the rat could hold onto the screen after being rotated from 0 to 90° was recorded to a maximum of 15 s (0–3). Rats were then tested for balance on a horizontal wooden rod, and the time lapse before falling off the rod was recorded to a maximum of 30 s (0–3). Finally, rats underwent a prehensile traction test, and the length of time that the rat could cling to a horizontal rope was recorded to a maximum of 5 s (0–3). Animals received a score for each of the three tests. The final score was the sum of the individual test scores, with 0 the best score, and 9 the worst score.

### Statistical Analysis

Statistical analysis was performed using Prism 8 (GraphPad Software, San Diego, CA, United States). Results are expressed as mean ± standard deviation (SD). Parametric values, including physiologic values, data from western blots, ELISA were compared between groups using one-way analysis of variance (ANOVA) with *post hoc* Tukey’s multiple comparison test, or a Student’s *t*-test (equal variance not assumed), according to the characteristics of each experiment. Statistical significance was defined as *p* < 0.05.

## Results

The 24 h survival rates were 75.0% (9/12) and 61.5% (8/13) for the ANXA1sp and vehicle treated groups, respectively. One rat in vehicle group and three in ANXAsp1 group died from acute cardiac failure during the early postoperative phase. Additionally, three rats died of severe spinal cord injury and one of severe brain injury, all in the vehicle treated group. Two rats in each group died of technical failures and were excluded from analyses.

**FIGURE 2:**
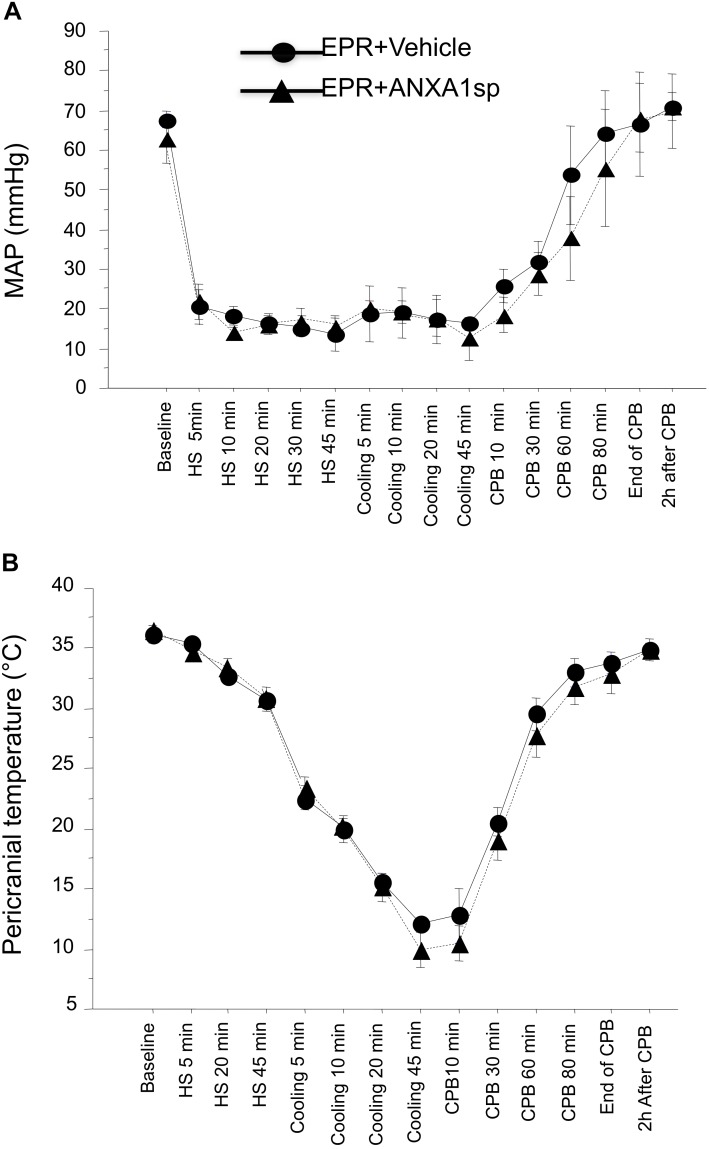
Time course of mean arterial pressure **(A)** and pericranial temperature **(B)** during the EPR experimental protocol for vehicle (control) and ANXA1sp-treated animals. Data presented as mean ± SD (*n* = 8–9/group).

Baseline and intraoperative physiological parameters were similar between groups and are shown in Figure 2A,B and Table 1. Profound systemic hypotension (MAP < 20 mmHg) occurred during rapid exsanguination and cardiac arrest (Figure 2A) and was associated with severe metabolic acidosis (Table 1). All animals underwent the same cooling and rewarming protocol (Figure 2B). Rats exhibited spontaneous hypothermia (from 37°C to 32°C) before the onset of systemic cooling (shock-induced hypothermia). Following induction of hypothermia, pericranial temperature rapidly dropped to 23°C at 5 min (rate of cooling 1.8°C/min) and to 15°C at 20 min (rate of cooling 0.85°C/min). After 45 min of profound hypothermia (10–15°C), animals were resuscitated and slowly rewarmed using CPB to 34–35°C over 80 min (rate of rewarming 0.4°C/min).

**Table 1:** Physiologic data.

Parameter	Group	Baseline	End of HS	End of cooling	CPB 30 min	CPB 80 min	End of CPB	2 h after CPB
pH	Vehicle	7.42 ± 0.06	7.19 ± 0.08	7.34 ± 0.09	7.39 ± 0.04	7.45 ± 0.09	7.29 ± 0.08	7.48 ± 0.07
	ANXA1sp	7.47 ± 0.04	7.17 ± 0.04	7.27 ± 0.05	7.35 ± 0.06	7.40 ± 0.1	7.32 ± 0.06	7.47 ± 0.05
PaO_2_ (mmHg)	Vehicle	202 ± 46	26 ± 8	46 ± 11^*^	291 ± 47	285 ± 89	240 ± 102	249 ± 77
	ANXA1sp	193 ± 26	25 ± 4	31 ± 3	313 ± 40	268 ± 65	253 ± 73	277 ± 131
PaCO_2_ (mmHg)	Vehicle	37.5 ± 6.9	54.8 ± 9.	52.2 ± 11.1	35.6 ± 6.6	34.0 ± 7.8	56.3 ± 13.4	42.2 ± 6.6
	ANXA1sp	32.9 ± 4.9	54.4 ± 4.7	57.2 ± 6.1	39.4 ± 5.0	39.3 ± 11.8	50.8 ± 5.1	41.6 ± 4.7
Hct (%)	Vehicle	38.00 ± 3.8	33.75 ± 2.4		18.63 ± 1.9	22.29 ± 2.4	23.57 ± 2.9	26.70 ± 4.4
	ANXA1sp	38.22 ± 5.5	35.22 ± 2.5		19.00 ± 1.7	23.78 ± 2.0	26.11 ± 5.9	32.50 ± 3.1
BE (mmol/L)	Vehicle	0.08 ± 3.6	-7.30 ± 2.9	1.26 ± 4.1	-3.03 ± 3.5	-0.94 ± 2.3	0.16 ± 2.6	6.86 ± 4.5
	ANXA1sp	0.47 ± 3.8	-8.57 ± 2.1	-1.69 ± 2.4	-3.46 ± 2.4	-1.31 ± 2.3	-0.51 ± 2.8	6.06 ± 1.8

*Results are shown as Mean ± SD; ^∗^p < 0.05 between groups. ANXA1sp, Annexin A1 short peptide; Hct, hematocrit; BE, base excess; End of HS, at the end of 45 min of hemorrhagic shock; End of cooling, at the end of 45 min cooling; End of CPB, at the end of cardiopulmonary bypass.*

### Systemically Administered ANXA1sp Traverses the Blood-Brain Barrier and Attenuates Neuroinflammation After EPR

**FIGURE 3:**
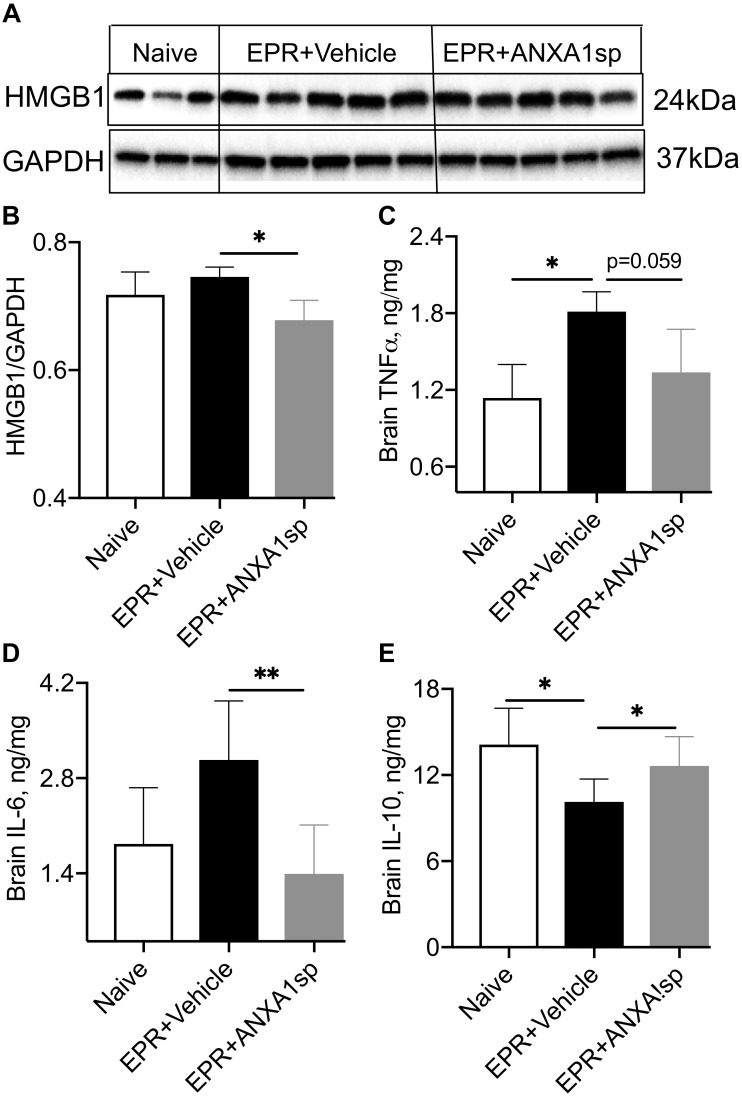
Modulation of indices of neuroinflammation after EPR by experimental group. **(A,B)** High mobility group protein B1 (HMGB1) expression in brain homogenates from ANXA1sp-treated animals were significantly reduced compared to vehicle (controls). **(C,D)** Cerebral levels of tumor necrosis alpha (TNFα) and interleukin 6 (IL-6) were increased in the vehicle controls, and reduced with systemic administration of ANXA1sp. **(E)** Cerebral interleukin 10 (IL-10) was significantly reduced after induction of profound hypothermia (EPR + vehicle) and restored with administration of ANXA1sp. Data presented as mean ± SD (*n* = 3–7/group), ^∗^*P* < 0.05 and ^∗∗^*P* < 0.01 compared to vehicle controls or naïve animals, analyzed by one-way ANOVA with Tukey’s multiple comparisons test. GAPDH, glyceraldehyde-3-phosphate dehydrogenase.

Relative abundance of HMBG1, a key initiator of neuroinflammation, was assessed by Western analysis in brain homogenates. We found a 10% (*p* < 0.05) attenuation in cerebral expression of HMGB1 in ANXA1sp compared to HS-EPR vehicle treated animals (Figure 3A,B). Moreover, brain levels of the pro-inflammatory cytokines IL-6 and TNFα were reduced by 55% (*p* < 0.01) and 27% (*p* = 0.059), respectively, with ANXA1sp treatment (Figure 3C,D). Conversely, brain levels of the anti-inflammatory cytokine IL-10 were increased by 25% (*p* < 0.05) in ANXA1sp-treated compared to vehicle controls at 24 h after EPR (Figure 3E).

Fluorescence-conjugated peptide (FITC-QAW) was detectable in all areas of brain parenchyma at 1 h following intravenous injection (Supplementary Figure 1).

### Regulation of Cell Death by ANXA1sp After EPR

**FIGURE 4:**
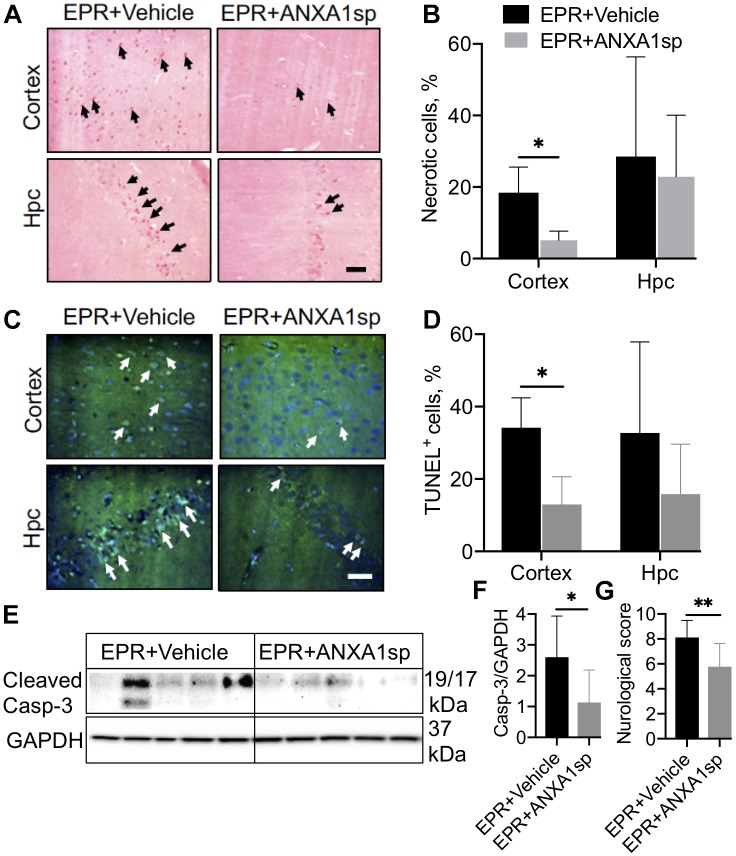
Necrosis and apoptosis in the CNS after exsanguination cardiac arrest/EPR and ANXA1sp or vehicle treatment. **(A,B)** Necrosis, detected by acid fuchsin-celestine blue, was reduced in the cerebral cortex of ANXA1sp treated rats. Arrowheads in panel A identify acidophilic neurons in the cortex and hippocampus (Hpc). **(C,D)** Terminal deoxynucleotidyl nick-end labeling (TUNEL) staining and quantification of apoptotic cells. Apoptosis was significantly reduced in the cerebral cortex of ANXA1sp-treated rats on postoperative day 1. Arrowheads in panel **(C)** identify pyknotic positive cells. Scale bar: 20 μm. **(E,F)** Western blot analysis of cleaved caspase-3 revealed significantly reduced levels in brain homogenates from ANXA1sp-treated animals compared to vehicle (controls). **(G)** Neurologic score (sensory-motor function) was significantly improved at postoperative day 1 in ANXA1sp rats. Data presented as mean ± SD (*n* = 5–7/group), ^∗^*P* < 0.05 and ^∗∗^*P* < 0.01 compared to vehicle (controls) analyzed by unpaired *t*-test **(B,D,F)** or Mann–Whitney *U*-test **(G)**.

Acid fuchsin-celestine blue staining revealed acidophilic neurons and possible necrosis in the cortex at 24 h after EPR in vehicle-treated animals, which were reduced by 72% (*p* < 0.05) following ANXA1sp treatment (Figure 4A,B). ANXA1sp treatment was also associated with a 62% (*p* < 0.05) reduction in TUNEL-positive cells in the cerebral cortex, but not in the hippocampus, at 24 h after EPR (Figure 4C,D). This was corroborated by a 56% (*p* < 0.05) reduction in cerebral expression of cleaved caspase-3 in ANXA1sp-treated animals (Figure 4E,F).

**FIGURE 5:**
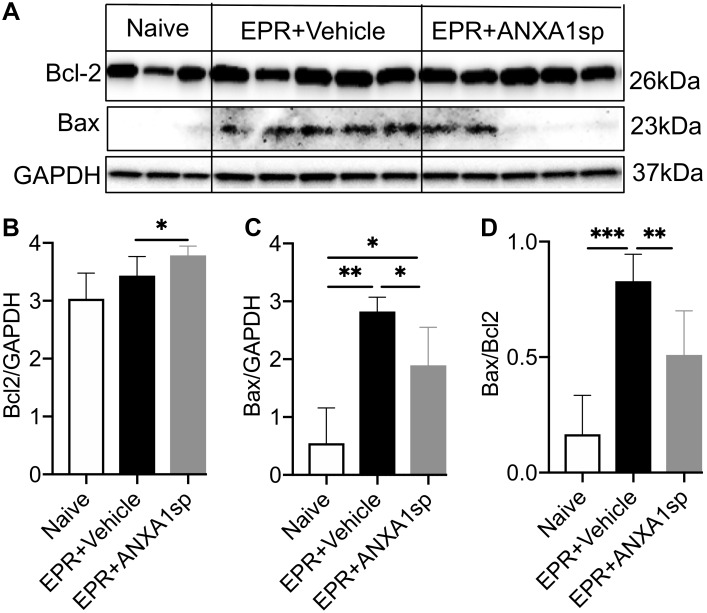
Western blot analysis of cerebral Bcl-2 and Bax levels after exsanguination cardiac arrest/EPR and ANXA1sp or vehicle treatment. Intensities of protein bands corresponding to Bcl-2 (26 kDa) **(A,B)** and Bax (23 kDa) **(A,C)** were measured using computer assisted densitometric analysis, normalized to the intensity of GAPDH and compared between experimental groups. ANXA1sp treatment resulted in significantly increased expression of the anti-apoptotic protein Bcl-2 **(B)**, reduced expression of the pro-apoptotic protein Bax **(C)**, with a corresponding reduction in the Bcl-2:Bax ratio **(D)**. Data presented as mean ± SD (*n* = 3–8/group), ^∗^*P* < 0.05, ^∗∗^*P* < 0.01, and ^∗∗∗^*P* < 0.001 compared to either naïve or vehicle controls analyzed by one-way ANOVA with Tukey’s multiple comparisons test.

We used Western analysis of brain homogenates to assess changes in ratios of death and survival factors. At 24 h, a modest but significant (10%, *p* < 0.05) increase in the relative abundance of the anti-cell death protein Bcl-2 was observed in ANXA1sp compared to vehicle-treated animals after HS-EPR (Figure 5A,B). Conversely, a marked 5-fold (*p* < 0.01) increase in expression of the pro-cell death protein Bax was seen after HS-EPR in vehicle-treated animals, with a significant 33% (*p* < 0.05) reduction following ANXA1sp treatment (Figure 5A,C). Consequently, the near 5-fold surge in Bax:Bcl-2 ratio detected after HS-EPR in vehicle-treated compared to naïve animals (*p* < 0.001) was attenuated by 38% (*p* < 0.01) with ANXA1sp treatment (Figure 5D).

### Modulation of SIRT3 Expression and SIRT3 Pathway by ANXA1sp

**FIGURE 6:**
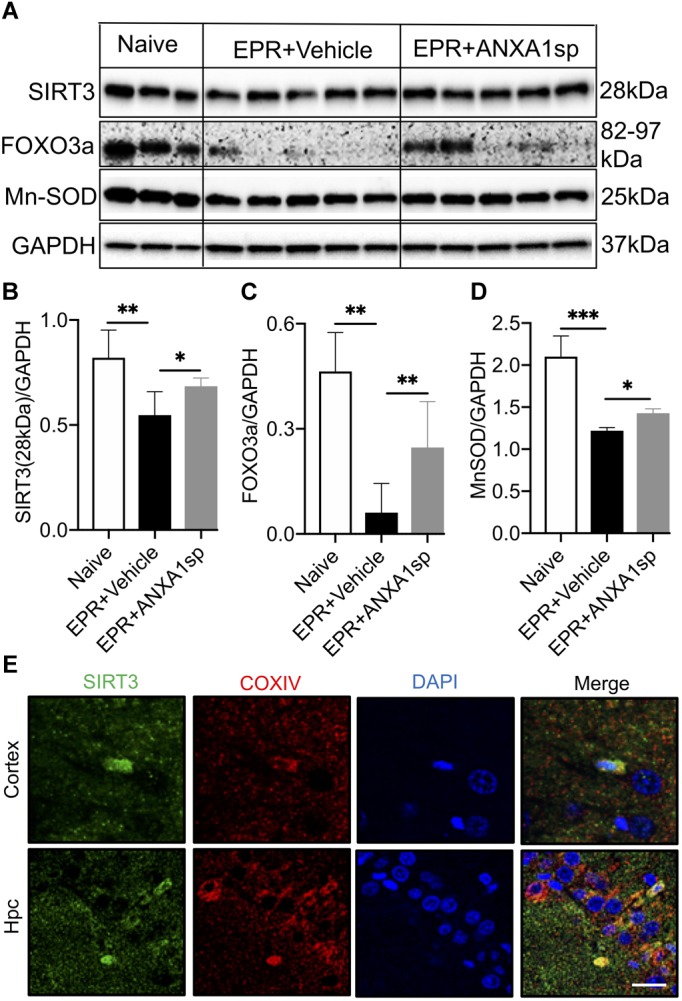
Cerebral expression of Sirtuin-3 (SIRT3) and downstream regulated proteins after exsanguination cardiac arrest/EPR and ANXA1sp or vehicle treatment. **(A,B)** A significant reduction in SIRT3 (28 kDa short, processed, active form) was seen after EPR + vehicle treatment compared to naïve animals, and was rescued in ANXA1sp-treated animals. This was accompanied by similar expression changes for the forkhead box O3a (FOXO3a) transcription factor **(C)**, known to transactivate the antioxidant enzyme manganese superoxide dismutase (MnSOD) **(D)**. GAPDH was used as loading control **(E)**. At 24 h postoperatively, colocalization of sirtuin-3 (SIRT3) with the mitochondrial protein cytochrome c oxidase complex IV (COXIV) in cortical and hippocampal slides from ANXA1sp-treated animals was visualized via double immunofluorescence staining and confocal microscopy. Data presented as mean ± SD (*n* = 3–7/group), ^∗^*P* < 0.05, ^∗∗^*P* < 0.01, and ^∗∗∗^*P* < 0.001 compared to either naïve or vehicle controls analyzed by one-way ANOVA with Tukey’s multiple comparisons test.

Western blot analysis in brain homogenates revealed a 33% (*p* < 0.01) reduction in relative abundance of the primary mitochondrial deacetylase SIRT3 (28 kDa isoform) in vehicle-treated HS-EPR compared to naïve animals, which was partially restored with ANXA1sp treatment (increased by 25%, *p* < 0.05) (Figure 6A,B). Increased SIRT3 expression with ANXA1sp treatment in both cortical and hippocampal cells was further confirmed by confocal microscopy, with SIRT3 immunoreactivity colocalizing with the mitochondrial protein COXIV (Figure 6E). We further measured expression of FOXO3a and MnSOD, two downstream targets of SIRT3. EPR resulted in an 87% (*p* < 0.01) reduction in relative abundance of the transcription factor FOXO3a and a 42% (*p* < 0.001) reduction its regulated antioxidant enzyme MnSOD compared to naïve animals, and were both partially restored with ANXA1sp treatment (a 4-fold increase in FOXO3a expression, *p* < 0.05; and a 18% increase in MnSOD expression, *p* < 0.05) (Figure 6A,C,D).

### ANXA1sp Promotes Autophagy Following EPR

**FIGURE 7:**
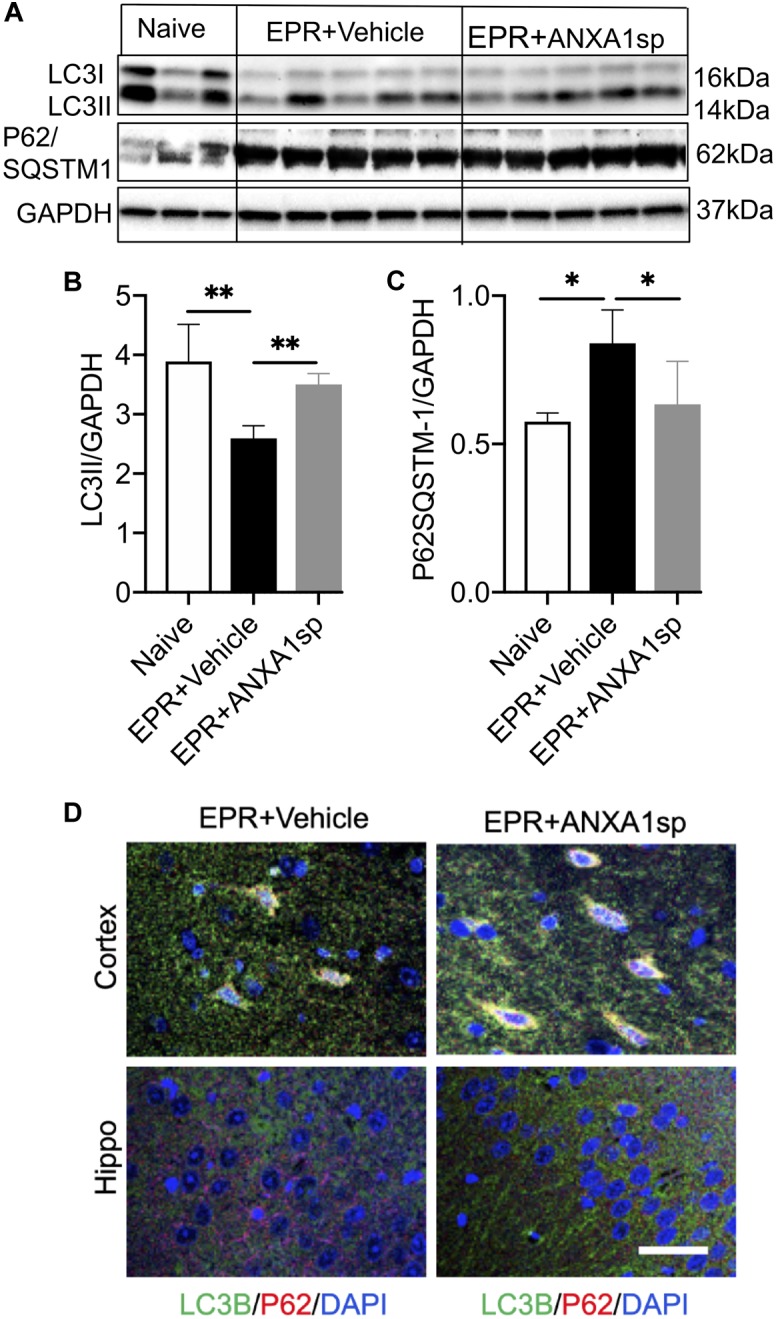
ANXA1sp promotes cerebral autophagy after exsanguinating cardiac arrest/EPR. The expression of LC3II and p62/SQSTM1 (sequestosome 1) was assessed by western blot and normalized to GAPDH **(A–C)**. **(D)** Colocalization of LC3B with p62 in cortical and hippocampal slides from animals treated with ANXA1sp and vehicle (controls) was visualized by double immunofluorescence staining and confocal microscopy. Increased number of LC3B positive/p62 positive puncta appears in the ANXA1sp-treated group. ^∗∗^*p* < 0.01; ^∗^*p* < 0.05.

Western blot was performed to detect the expression of autophagy associated proteins in brain homogenates. Compared to vehicle, ANXA1sp treatment was associated with a 35% (*p* < 0.01) increased expression of LC3II and a 25% (*p* < 0.05) reduced expression of p62/SQSTM1 (Figure 7A,B,C). In addition, we detected expression of LC3B by immunohistochemistry, with LC3B and p62 colocalizing in cortical cells (Figure 7D).

### Neurologic Outcome After EPR and ANXA1sp Treatment

Finally, we evaluated neurologic changes at 24 h after EPR and ANXA1sp treatment. Compared to vehicle controls, ANXA1sp-treated animals had significantly lower neurologic severity scores, showing improved sensory-motor functions (including processing involving retrosplenial and posterior parietal cortex, Figure 4G).

### Systemically Administered ANXA1sp Also Attenuates Myocardial Inflammation

To evaluate the effects of ANXA1sp in organs that are not shielded by the blood-brain barrier, we assessed pro-inflammatory cytokines in left ventricular myocardial homogenates between groups. At 24 h after EPR, heart levels of IL-6 and TNFα were reduced in ANXA1sp-treated animals by 10% (*p* < 0.05) and 17% (*p* = 0.054), respectively (Supplementary Figure 2).

## Discussion

In this study, we used a novel experimental model of acute neuroinflammation triggered by exsanguinating cardiac arrest and treated by emergent preservation and resuscitation (EPR) to test the neuroprotective effects and mechanisms of ANXA1sp administration. Together with our previous findings that ANXA1sp exerts pro-resolving effects in a rat model of CPB with global ischemia-reperfusion via deep hypothermic circulatory arrest (Zhang et al., 2017), we continue to illustrate how this small peptide impacts neuroinflammation. Here, we show that ANXA1sp treatment is superior to induction of profound hypothermia (EPR) alone by increasing cortical cell viability, promoting a favorable expression of pro-survival (Bcl-2) versus pro-death (Bax) factors in the brain, reducing caspase-3 activation, and overall improving neurological performance at 24 h after EPR.

High mobility group box 1 is a key endogenous danger associated molecular pattern which acts as a mediator of neuroinflammation resulting from a variety of conditions such as cerebral ischemia-reperfusion (Kim et al., 2006; Yang et al., 2011), septic shock, and traumatic brain injury (Parker et al., 2017). HMGB1 is actively released by neurons and glial cells upon inflammasome activation, and in turn activates two pattern recognition receptors on target cells (TLR4 and RAGE) (Gao et al., 2012), leading to NF-κB mediated production of pro-inflammatory cytokines (Paudel et al., 2018). HMGB1 also plays a pivotal role in BBB disruption (He et al., 2012; Festoff et al., 2016; Yang et al., 2018), either directly via cytokine mediated activation of metalloproteinase or via disruption of tight junctions (Gloor et al., 2001; Utech et al., 2010), although the precise mechanism remains elusive. Nonetheless, altered BBB permeability is known to then amplify neuroinflammation and neuronal excitation (Frank et al., 2015, 2016). We provide primary evidence that HS and exsanguinating cardiac arrest treated with induction of profound hypothermia and EPR elicit neuroinflammation. Further, we show ANXA1sp treatment to favorably modulate the neuroinflammatory response, by attenuating the HS-EPR induced increase in cerebral levels of the proinflammatory alarmin HMGB1 and altering the balance of pro-inflammatory (IL-6 and TNFα) and anti-inflammatory cytokines (IL-10) (Figure 3A–E). This is consistent with the pleiotropic immunomodulatory functions of IL-10, to polarize the inflammatory system toward an anti-inflammatory phenotype (Ip et al., 2017), aiding in the resolution of neuroinflammation (Garcia et al., 2017). The pro-resolving effects were accompanied by improved cell survival (Figure 4A–E) and neurological function (Figure 4G). This study builds on our previous findings demonstrating beneficial effects of ANXA1sp on neuroinflammation, microglial activation, NF-κB activation and postoperative neurocognitive performance following global I/RI associated with deep hypothermic circulatory arrest (Zhang et al., 2017). While our experimental model is complex, sequentially involving HS, warm cardiac arrest, induction of profound hypothermia followed by rewarming and resuscitation, the protective effects of ANXA1sp recapitulate those seen in other experimental models of cerebral I/RI (Relton et al., 1991; Gavins et al., 2007; Smith et al., 2015; Vital et al., 2016).

Systemically administered ANXA1sp was also associated with anti-inflammatory effects in the heart (reduced levels of IL-6 and TNFα) following HS-EPR. Consistent with previous reports in experimental models of regional myocardial I/RI (La et al., 2001; Qin et al., 2015), these results support the broader therapeutic roles attributed to ANXA1 and its peptide mimetics in reducing systemic inflammation and conferring organ protection from a variety of insults involving IR/I.

To better understand the added neuroprotective effects and mechanisms of action of ANXA1sp in the setting of HS-EPR with profound hypothermia, we focused on SIRT3, a mitochondrial protein deacetylase known to be widely and abundantly expressed in most cell types in the CNS (Kim et al., 2011) and differentially regulated in brain regions and across stages of development in the rat (Sidorova-Darmos et al., 2014). SIRT3 is part of the silent information regulator of transcription (SIRT) family of NAD^+^-dependent protein deacetylases, which have emerged as key regulators of metabolic and energy stress-response pathways including neuroinflammation (Someya et al., 2010; Lee et al., 2013; Hattori et al., 2015; Liu L. et al., 2015; Yuan et al., 2016), but also systemic inflammation (Preyat and Leo, 2013; Vachharajani et al., 2014; Liu T. F. et al., 2015; Traba et al., 2015). Notably, SIRT3 exerts neuroprotective effects. For instance, SIRT3 overexpression rescues mutant SOD1-induced neuronal cell death (Song et al., 2013), increases neuronal lifespan under mitochondrial oxidative stress (Weir et al., 2012), protects against excitotoxic injury (Kim et al., 2011), mediates adaptive neuronal responses (resistance to oxidative stress, apoptotic cell death) to *in vitro* bioenergetic, oxidative and excitatory stress (Cheng et al., 2016), and protects mice against noise-induced hearing loss *in vivo* (Someya et al., 2010). Specifically, SIRT3 has been shown to have a major involvement in CNS ROS metabolism by regulating FOXO3a transcription factor and contributing to transactivation of key antioxidant enzymes including MnSOD and catalase (Rangarajan et al., 2015). Together, even though results from rodent studies are not completely consistent (Novgorodov et al., 2016), most data suggest that SIRT3 expression is vital for neuronal survival and suppression of neuroinflammation following physical stressors. In this study, we noted that relative abundance of SIRT3 – the 28 kDa processed active isoform, known to possess deacetylase activity (Schwer et al., 2002; Sundaresan et al., 2008) – as well as FOXO3a and MnSOD, were all reduced in brain homogenates following HS-EPR, consistent with previous reports in experimental models of cerebral I/R (Zhao et al., 2018), but were rescued with systemic administration of ANXA1sp (Figure 6). HS, ischemia-reperfusion, and EPR incite neuroinflammation, and SIRT3-mediated upregulation of FOXO3a-dependent antioxidant gene expression could be implicated in the attenuation of ROS neurotoxicity associated with acute neuroinflammation and activation of microglia. This may translate into attenuation of secondary brain injury and decreased cerebral edema. Along with downregulation of pro-apoptotic proteins, these findings suggest that ANXA1sp treatment offers an element of neuroprotection.

Modulation of protein acetylation is emerging as a therapeutic strategy to create a pro-survival and anti-inflammatory phenotype in various shock states, including lethal HS (Li and Alam, 2011). Intriguingly, promoting histone acetylation via administration of the histone deacetylase (HDAC) inhibitor valproic acid has been shown to decrease neuronal apoptosis, neuroinflammation, and brain lesion size while improving neural plasticity and resulting in faster neurocognitive and neurologic recovery in pre-clinical models of traumatic brain injury, HS, and polytrauma (Nikolian et al., 2017, 2018; Chang et al., 2019). An imbalance in histone acetyltransferase/deacetylase activity has been reported in both HS (Lin et al., 2006) and neurodegeneration (Rouaux et al., 2003). Valproic acid administration results in epigenetic and posttranslational modifications that induce differential gene expression (Dekker et al., 2014; Bambakidis et al., 2016), metabolic changes (Hwabejire et al., 2013) and alterations of the proteome. It appears that promoting acetylation of histones and other target transcription factors through the use of HDAC inhibitors (like valproic acid), as well as promoting mitochondrial protein deacetylation via induction of SIRT3 (in this study using ANXA1sp) can both exert neuroprotective effects in the setting of lethal HS. The precise mechanisms by which modulation of protein acetylation results in attenuation or resolution of neuroinflammation remain to be elucidated.

**FIGURE 8:**
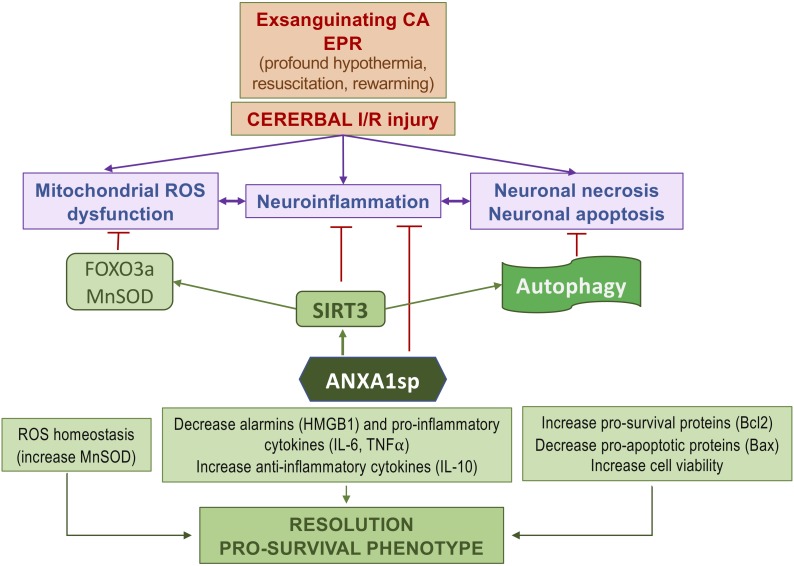
Simplified diagram of the protective effects of ANXA1sp following cerebral ischemia-reperfusion injury associated with experimental exsanguinating cardiac arrest (CA) and emergent preservation and resuscitation (EPR).

We found that ANXA1sp treatment promoted cerebral autophagy following EPR. This was accompanied by increased SIRT3 protein expression, reduced cell apoptosis and improved neuro severity scores. Importantly, upregulation of SIRT3 in response to ANXA1sp treatment was associated with increased expression of LC3II and a reduced level of p62/SQSTM1, indicating increased autophagic flux (Figure 7). These results are consistent with previous reports implicating SIRT3 as an activator of autophagy in neuronal cells (Dai et al., 2017; Yan et al., 2018) and macrophages (Liu et al., 2018) under various stress conditions *in vitro*, and *in vivo* studies showing sirtuin activation to be protective in spinal cord injury, brain trauma, and cerebral ischemia (He et al., 2017; Zhao et al., 2017). A schematic representation of the relationships between ANXA1sp, the SIRT3 pathway and neuroinflammation in the context of cerebral I/RI is shown in Figure 8.

Although the therapeutic effects of hypothermia in HS in general, and those of EPR for exsanguinating cardiac arrest in particular, have been previously reported in large animal experiments (Alam et al., 2002, 2004, 2005, 2006a,b; Chen et al., 2005; Wu et al., 2006), benefits of the clinically realistic rat model described in this study include lower costs to test efficacy of selective pharmacological neuroprotective interventions (such as the pro-resolving ANXA1sp) in enhancing clinical outcomes after EPR, as well as availability of molecular biology tools to understand the mechanisms at play. To increase clinical relevance, we modified previously described rodent models of EPR (Drabek et al., 2007a,b) in several ways: first, we introduced a prolonged period of HS followed by induction of fibrillatory cardiac arrest; second, we rapidly induced profound hypothermia via aortic flushing of a relatively low volume (1.5–2 fold the estimated blood volume) of ice-cold saline-based solution using a light weight cycling system at 20 ml/min; finally, we re-transfused salvaged autologous blood during resuscitation, thereby avoiding additional confounding effects from exposure to allogeneic blood transfusions. In this severe model, administration of a pro-resolving ANXA1 bioactive peptide during EPR was superior to EPR alone in attenuating neuroinflammation, increasing pro-survival protein expression and autophagic flux.

Several limitations of this study follow. HS was achieved by closely controlled exsanguination of blood, which was salvaged and re-transfused. Although we designed the model to closely mimic the clinical scenario, HS inevitably requires surgical bleeding control and transfusion of allogeneic blood products, which would significantly amplify the inflammatory response. Rats serve as imperfect surrogates for human subjects, and only male young adult animals were used. An injured but un-resuscitated control group was not used, as the survival without resuscitation is extremely poor. Instead, we used a naïve uninjured control group, to allow assessing whether ANXA1sp effects are restorative or *de novo*. We have not measured directly markers of resolution of inflammation (neutrophil apoptosis/efferocytosis, functional macrophage switches), or microglial activation. However, microglial activation was attenuated by ANXA1sp treatment in our previously reported rat deep hypothermic circulatory arrest study (Zhang et al., 2017). This study focuses on ANXA1sp-induced upregulation of the SIRT3 pathway, and thus we have not explored the traditional mechanisms implicated in the pro-resolving effects of Annexin A1 and its bioactive peptides (via the FPR2/ALX receptor pathway). The neuroinflammatory response to hypothermic cardiac arrest is not uniform, displaying important spatio-temporal differences between brain regions (Drabek et al., 2015). Our investigation focused on the cortex and hippocampus as two vulnerable brain regions, but other areas displaying robust early neuroinflammatory responses (e.g., the striatum) were not studied. Temporally, our observation period was limited to 24 h, and longer-term evaluations are required to fully assess ANXA1sp’s protective effects, including neurocognitive tests. Although previous reports from experimental focal cerebral I/RI in the rat suggest neutrophil accumulation in the infarct area (which correlates closely with cortical lesion size) to be maximal at 24 h post-reperfusion (Zhang et al., 1994), it should be noted that our results may represent a delay rather than elimination of neuronal death in the selectively vulnerable brain regions studied.

## Conclusion

Using a clinically relevant rodent model of lethal HS and EPR-induced acute neuroinflammation, we show that administration of a pro-resolving ANXA1 peptide mimetic results in a pro-survival phenotype with attenuated cortical cell death, reduction of several common functional biomarkers of neuroinflammation (HMGB1, IL-6, and TNFα), and improved early neurological outcomes, compared to induction of profound hypothermia (EPR) alone. The protective effects were associated with increased expression of SIRT3 and corresponding upregulation of the FOXO3a-MnSOD antioxidant pathway. In addition, we note increased cerebral autophagy in ANXA1sp treated animals. Further studies need to refine the mechanisms involved and evaluate the use of SIRT3-activating drugs to resolve acute neuroinflammation and attenuate the uncontrolled production of ROS by activated microglia, as well as the long-term benefits of the proposed therapeutics.

## Data Availability

All datasets generated for this study are included in the manuscript and/or the Supplementary Files.

## Ethics Statement

This study was carried out in accordance with the recommendations of the National Institutes of Health for animal care (Guide for the Care and Use of Laboratory Animals, Health and Human Services, National Institute of Health Publication No. 86-23, revised 1996). The protocol was approved by the Duke University Animal Care and Use Committee.

## Author Contributions

ZZ, QM, J-KS, and MP designed and performed the research. TV, CL, JM, and NT contributed new reagents and analytic tools. ZZ, QM, QQ, and MP analyzed the data. QM, ZZ, and MP wrote the manuscript. QQ, JM, and NT provided the critical edits to the manuscript. All authors read and approved the final draft of the manuscript.

## Conflict of Interest Statement

ZZ, QM, NT, and MP are co-inventors on patents for the use of Annexin A1 peptides for activation of sirtuins and to attenuate neuroinflammation. NT is Associate Editor for Frontiers in Immunology. The remaining authors declare that the research was conducted in the absence of any commercial or financial relationships that could be construed as a potential conflict of interest.

## Abbreviations

ANXA1sp: Annexin A1 short peptide
CPB: cardiopulmonary bypass
EPR: emergency preservation and resuscitation
HMGB1: high mobility group box 1
HS: hemorrhagic shock
LC3-II: microtubule-associated protein 1A/1B-light chain 3-phosphatidylethanolamine conjugate
MnSOD: manganese superoxide dismutase
p62/SQSTM1: sequestosome-1
ROS: reactive oxygen species
SIRT3: sirtuin-3
