# Supplementary material for: Annexin A1 Bioactive Peptide Promotes Resolution of Neuroinflammation in a Rat Model of Exsanguinating Cardiac Arrest Treated by Emergency Preservation and Resuscitation
Source: Front Neurosci. 2019 Jun 14;13:608. doi: 10.3389/fnins.2019.00608 (PMC6587399; doi:10.3389/fnins.2019.00608)
Supplement: Supplementary file 1 [file Data_Sheet_1.PDF]

# *Supplementary Material*

## 1 Supplementary Figures

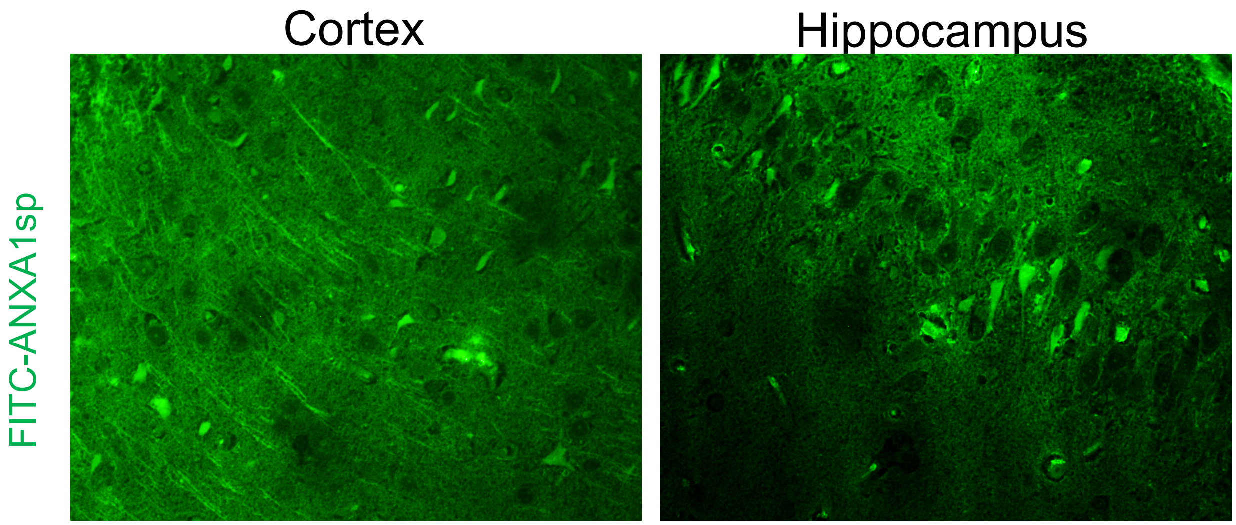


**Supplemental Figure 1.** Delivery of ANXA1sp across the blood-brain barrier of rat. Representative fluorescence microscopic images (Leica DM IRB, Germany equipped with a 20×/0.4 PH objective) of cortical and hippocampal slides from rats 1h following intravenous injection of fluorescein isothiocyanate (FITC)-conjugated ANXA1sp (MW 905.98). Diffuse green fluorescence is seen in all areas of brain parenchyma, including cortex and hippocampus, and various cell types (neurons and endothelial cells).

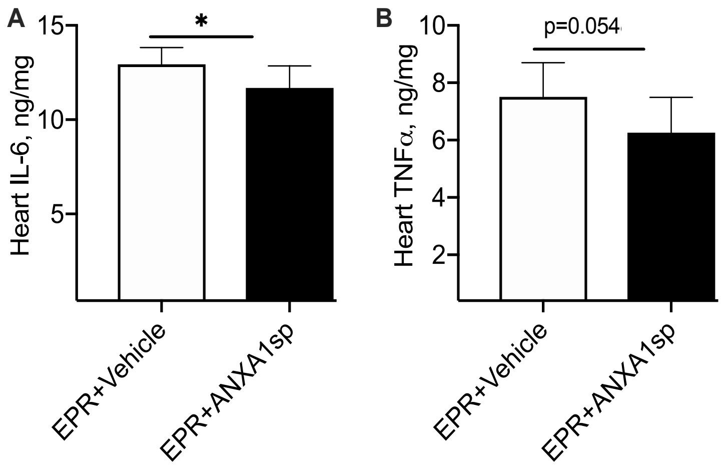


**Supplemental Figure 2.** Systemic administration of ANXA1sp attenuates myocardial inflammation at 24h following exsanguinating cardiac arrest and EPR. Data presented as mean ± SD (n = 5-7/group), *P<0.05, compared to vehicle controls, analyzed by Student's *t*-test.
